# Quantifying the Predictability of Visual Scanpaths Using Active Information Storage

**DOI:** 10.3390/e23020167

**Published:** 2021-01-29

**Authors:** Patricia Wollstadt, Martina Hasenjäger, Christiane B. Wiebel-Herboth

**Affiliations:** Honda Research Insitute Europe GmbH, Carl-Legien-Str. 30, 63073 Offenbach/Main, Germany; martina.hasenjaeger@honda-ri.de (M.H.); christiane.wiebel@honda-ri.de (C.B.W.-H.)

**Keywords:** eye tracking, information theory, active information storage, scanpath

## Abstract

Entropy-based measures are an important tool for studying human gaze behavior under various conditions. In particular, gaze transition entropy (GTE) is a popular method to quantify the predictability of a visual scanpath as the entropy of transitions between fixations and has been shown to correlate with changes in task demand or changes in observer state. Measuring scanpath predictability is thus a promising approach to identifying viewers’ cognitive states in behavioral experiments or gaze-based applications. However, GTE does not account for temporal dependencies beyond two consecutive fixations and may thus underestimate the actual predictability of the current fixation given past gaze behavior. Instead, we propose to quantify scanpath predictability by estimating the active information storage (AIS), which can account for dependencies spanning multiple fixations. AIS is calculated as the mutual information between a processes’ multivariate past state and its next value. It is thus able to measure how much information a sequence of past fixations provides about the next fixation, hence covering a longer temporal horizon. Applying the proposed approach, we were able to distinguish between induced observer states based on estimated AIS, providing first evidence that AIS may be used in the inference of user states to improve human–machine interaction.

## 1. Introduction

The analysis of visual scanning behavior provides a rich source of information in the investigation of observers’ cognitive processes or states [1,2,3,4,5]. One aspect of visual scanning behavior are *scanpaths*, which denote sequences of consecutive fixations, where a fixation is a period of little to no eye movement that allows for gathering of visual information [6]. The analysis of scanpaths has gained renewed interest in recent years, for example, to study cognitive function [7,8], personality traits [9,10], or as marker in gaze-based applications [11]. In particular, information-theoretic measures have become a popular tool for studying cognitive function through the analysis of human gaze behavior [5,8,12,13,14,15,16,17]. A commonly used measure is the *(gaze) transition entropy* (GTE) [17], which uses a conditional Shannon Entropy [18] to describe the regularity of transitions between fixations of pre-defined areas of interest (AOI) [5]. GTE considers sequences of fixations, so-called scanpaths, under the assumption that scanpaths can be modeled as Markov chains of order one, and is calculated as the entropy of the transitions between two consecutive fixations. Low GTE—and thus a low remaining uncertainty about the location of the next fixation given the previous one—thereby is interpreted as a high predictability of the next fixation [5]. GTE has been applied in various studies (see [5] for a review), which have shown that changes in GTE are associated with higher task demand [13,19,20], increased anxiety [21,22,23], or sleep deprivation [14].

Despite the popularity of information-theoretic measures, alternative approaches have been employed in the analysis of scanpaths (e.g., [24,25,26,27]). One example is the work by Coutrot et al. [27], who demonstrated an alternative approach to modeling scanpath data using Hidden Markov Models (HMM) in order to decode different task or observer characteristics. However, similar to GTE, their approach does not test or account for the influence of past fixations beyond the immediate last fixation. This view has however been challenged by a number of recent studies that found evidence for the importance of incorporating *long-range* temporal information when analyzing and modeling eye movement data [7,25,28]. Here, long-range denotes any dependency of the location of the current fixation on a fixation further in the past than the immediate last fixation.

Hayes et al. [25], for example, introduced the Successor Representation (SR model) to the analysis of scanpaths, which uses an algorithm from reinforcement learning to represent sequential gaze data in a temporally extended fashion. More precisely, the SR model stems from temporal difference learning [29,30] and incorporates among others a temporal parameter that defines the time span for which an observation (fixation) influences the model outcome. The authors found that up to 40% of the variance in viewer intelligence, working memory capacities, and speed of processing could be explained based on differences in scan patterns that were individually modelled with the SR model [7,25], as well as to some extent variances in ADHD scores (up to 50%), autism quotients (up to 30%), and dyslexia scores (up to 25%) [31]. Wiebel-Herboth et al. [28] found that an SR model had a significantly higher predictive power when classifying single participants based on their scanpaths in a visual comparison task compared to a simple transition matrix model that considered only the immediate last fixation. Moreover, Hoppe et al. [32] were the first to provide quantitative evidence that humans are capable of planning eye movements beyond the next fixation by exploiting a priori knowledge about the task and stimulus in their experimental paradigm. While the authors did not provide a generic method for modeling scanpaths, their results provide a potential mechanism for the generation of long-range correlations in gaze behavior. Taken together, these results suggest that longer temporal dependencies in scanpaths may exist and be informative about their underlying cognitive processes and thus should be included in the modeling process.

However, entropy-based measures commonly applied, e.g., GTE [5,16,17], typically only take into account information contained within the immediate past fixation when quantifying the regularity of eye movements. When GTE was first introduced, Krejtz and colleagues [16,17] adopted the procedure by Julian and Mondal [33] for testing the Markov chain of order one assumption underlying the GTE computation. In their experiment, they found that in most but not all cases the assumption was valid. To our knowledge, such a validation procedure has not yet become a standard procedure in the entropy-based gaze analysis literature (for a review, see [5]). In cases where the order-one Markov chain assumption is violated, longer temporal dependencies are not accounted for in current information-theoretic approaches to scanpath analysis. As a result, if such temporal dependencies existed in a scanpath, the GTE would presumably underestimate its overall predictability.

Alternative modeling approaches, such as the SR model, also come with drawbacks. Most importantly, the model parameters have to be defined ad-hoc and cannot be learned in a data-driven fashion. This entails the risk of a circular argumentation if no external optimization criterion can be defined. Furthermore, the model parameters are not interpretable in a straightforward way, which limits the explanatory power of the approach. Thus, there is still a need for methods of scanpath modeling that can integrate both spatial and temporal information in the data [34].

To this end, we here propose a novel approach to the information-theoretic analysis of scanpath data, which is able to measure predictability in a scanpath while accounting for temporal dependencies of arbitrary order: we propose to estimate active information storage (AIS) [35] from scanpaths, which measures the predictability of a sequence as the mutual information between the sequences’ past and its next state. In particular, the relevant past is modeled as the collection of all past variables that provide information about the next value and can be identified using novel estimation procedures that optimize the past state in a data-driven fashion [36,37,38].

AIS has been successfully applied in a variety of disciplines to measure predictability of time series [39,40,41,42,43,44]. In the context of scanpath analysis, we believe AIS can provide several benefits at once: (1) It implicitly tests the order-one Markov chain assumption, as it provides the optimized past state for a given data sample. As such, it can provide direct evidence for whether fixations beyond the last fixation have a predictive value. (2) The length of the optimized past state is directly interpretable. That is, the optimization finds the temporal horizon over which past fixation(s) are informative about and thus has the potential to support the generation of explanatory hypothesis. (3) AIS allows for an individually optimized computation of predictability that may be of greater usefulness in gaze-based applications, e.g., driver assistance [11,14,45]. In sum, we argue that AIS may be applied to quantify the predictability of a scanpath, in particular, while information provided by fixations beyond the immediate fixation can be detected and accounted for.

In the following, we introduce AIS together with the necessary information-theoretic background and describe its estimation from scanpath data. As a proof of concept, we estimate AIS from scanpath data recorded in a visual comparison task under two different experimental conditions that induced different observer states. The contributions of our paper are the following: we provide the first application of AIS in the analysis of scanpath data. The AIS estimation revealed that most participants showed temporal correlations beyond the last fixation in the data. We demonstrate that changes in predictability quantified by AIS reflect changes in experimental condition that induced different observer states. Moreover, we show that the experimental condition can be successfully decoded from AIS estimates, indicating that AIS is a promising feature for identifying user states. The decoding based on AIS estimates resulted in a significantly higher accuracy than decoding from GTE, which indicates that long-term information captured by AIS but not GTE adds relevant information about the observer state in our experiment.

## 2. Materials and Methods

### 2.1. Information-Theoretic Preliminaries

Formally, we consider a scanpath as realizations $(x_{1},\ldots ,x_{t},\ldots ,$$x_{N})$, $x_{t}\in A_{X_{t}}$ of a stationary random process $X=(X_{1},X_{2},\ldots ,X_{t},$$\ldots ,X_{N})$, where a random process is a collection of random variables, *X*, ordered by an integer $t\in \left\{1,\ldots ,N\right\}\subseteq N$. As a shorthand, we write $p(x_{t})$ for the probability, $p(X_{t}=x_{t})$, of variable $X_{t}$ taking on the value $x_{t}\in A_{X_{t}}$, and $p(x_{t}|y)$ for the conditional probability of $X_{t}$ taking on the value $x_{t}$ if the outcome *y* of a second variable, *Y*, is known.

The Shannon entropy [18] is then defined as

(1) $$H\left(X\right)=-\sum_{x\in A_{X}}p\left(x\right)\log p\left(x\right)$$

and quantifies the expected uncertainty associated with the random variable *X* or the amount of information to be gained when observing outcomes of *X*. The conditional entropy is then the average information or uncertainty remaining in *X* if the outcome of *Y* is known:

(2) $$H\left(X|Y\right)=-\sum_{x\in A_{X},y\in A_{Y}}p\left(x,y\right)\log p\left(x|y\right).$$

Based on these definitions, we define the mutual information (MI) as the average amount of information one variable, *X*, provides about a second variable, *Y*,

(3) $$\begin{array}{cc} I(X;Y)= & H(X)-H(X|Y)=H(Y)-H(Y|X)\\ = & \sum_{x,y}p\left(x,y\right)\log\frac{p(x|y)}{p(x)}. \end{array}$$

**Figure 1:**
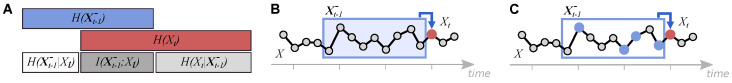
(**A**) Relationship between entropy and conditional entropy, *H*, and mutual information *I* of two non-independent random variables $X_{t}$ and $X_{t-1}^{-}$ (adapted from [46]). Here, the conditional entropy corresponds to the gaze transition entropy (GTE). (**B**) Active information storage (AIS) quantifies the predictability of the value of time series *X* at time *t*, $X_{t}$ (red marker), from its immediate past state, $X_{t-1}^{-}$ (blue box). (**C**) Non-uniform embedding representing the past state of time series, *X*, as a selection of past variables (blue markers) up to a maximum lag $k_{max}<t$, which carry significant information about the next value, $X_{t}$ (red marker).

The MI quantifies the information *X* provides about *Y* and vice versa; it is zero for independent variables (p(x,y)=p(x)p(y)) or if either H(X) or H(Y) is zero, i.e., there is no information to share. The MI is bound from above by the entropy of both variables involved, 0≤I(X;Y)≤H(X),H(Y) (Figure 1A).

### 2.2. Active Information Storage (AIS)

AIS [35] quantifies how much information a processes’ past state $X_{t-1}^{-}$ contains about its next value $X_{t}$ and thus measures the average predictability of $X_{t}$ from its immediate past [35,47] (Figure 1B). AIS is calculated as the MI between $X_{t-1}^{-}$ and $X_{t}$,

(4) $$\begin{array}{cc} AIS(X_{t}) & =I\left(X_{t-1}^{-};X_{t}\right)=H\left(X_{t}\right)-H\left(X|X_{t-1}^{-}\right)\\ \; & =\sum_{x_{t},x_{t-1}^{-}}p\left(x_{t},x_{t-1}^{-}\right)\log\frac{p(x_{t}|x_{t-1}^{-})}{p(x_{t})}, \end{array}$$

where the past state $X_{t-1}^{-}$ is defined as a collection of random variables up to a maximum lag $k_{max}$ (see also the next section),

(5) $$X_{t-1}^{-}=\left\{X_{t-1},\ldots ,X_{t-t_{l}},\ldots ,X_{t-k_{max}}\right\}.$$

AIS is low for processes with highly random transitions and high for processes that visit many different states in a regular fashion [35,47]. Formally, $0\leq AIS\left(X_{t}\right)\leq H\left(X_{t}\right),H\left(X_{t-1}^{-}\right)$, i.e., AIS is zero for processes with no memory such that they are completely random, and the AIS is upper bounded by the entropy of the past state and entropy of the next value of a process.

#### Relationship between GTE and AIS

GTE measures the remaining uncertainty in a fixation, given knowledge of the previous fixation as a conditional entropy, $H(X_{t}|X_{t-1})$. Hence, for past states of length one, AIS and GTE are complementary, i.e., $H\left(X_{t}\right)=I\left(X_{t};X_{t-1}\right)+H\left(X_{t}|X_{t-1}\right)$ (Equation (4) and Figure 1A). However, for processes that do not fulfill the Markov condition, $p(X_{t}|X_{t-1},\ldots ,X_{t-l})$ = $p(X_{t}|X_{t-1})$, i.e., processes that are not sufficiently described by a Markov chain of order one, GTE may underestimate the actual predictability of the next state from the whole *relevant* past of *X* (see also the next section). Furthermore, both measures differ in their interpretation—while GTE measures the remaining uncertainty in the next fixation, AIS, as a MI, measures how much information the past provides about the next fixation. The latter thus provides a more direct measure of predictability [47,48].

### 2.3. Estimating AIS from Scanpath Data

#### 2.3.1. Optimization of Past States

To estimate AIS in practice, we first have to define the past state $X_{t-1}^{-}$ such that it contains all relevant information stored in the past of *X* about $X_{t}$ [35]. Formally, we want to define $X_{t-1}^{-}$ such that $p\left(X_{t}|X_{t-1},\ldots ,X_{t-l}\right)=p\left(X_{t}|X_{t-1}^{-}\right)$. In other words, the next value, $X_{t}$, is conditionally independent of all past variables, $X_{t-l},l>k_{max}$, given $X_{t-1}^{-}$. Non-optimal choices for $X_{t-1}^{-}$ may lead to an underestimation of AIS if not all relevant information is covered by $X_{t-1}^{-}$, or they may lead to artificially inflated AIS values if too many variables are included, leading to an under-sampling of the past state.

Here, we find $X_{t-1}^{-}$ through a non-uniform embedding procedure [36,49] that selects a subset of variables from all past variables up to a maximum lag, $k_{max}$ (Figure 1C),

(6) $$X_{t-1}^{-}=\left\{X_{t-k}\right\},\;k\in \left[1,k_{max}\right].$$

More formally, our goal is to identify the minimal set of variables, $X_{t-1}^{-}$, that provides maximum information about $X_{t}$, $\max_{X_{t-1}^{-}}I\left(X_{t-1}^{-};X_{t}\right)$. We optimize $X_{t-1}^{-}$ using a greedy forward-selection approach implemented in [37], which iteratively includes variables $X_{t-k}$ if they provide significant, additional information about $X_{t}$, conditional on all already selected variables, i.e., $\max_{X_{t-k}}I\left(X_{t-k};X_{t}|X_{t-1}^{-}\right)$ (see [36,37,38,49] for more details). The implementation of the forward-selection approach uses a hierarchical permutation testing scheme to handle estimator bias, while controlling the family-wise error rate during repeated testing [37,38]. Using statistical testing for the inclusion of variables further provides an automatic stopping criterion for construction of the past state. The implementation of the AIS estimation procedure used is freely available as part of the IDTxl open-source Python package [37], https://github.com/pwollstadt/IDTxl.

#### 2.3.2. Estimating AIS from Discrete Scanpath Data

After optimizing $X_{t-1}^{-}$, we estimate AIS from scanpath data using plug-in estimators [50] that are known to exhibit a bias due to finite sampling (e.g., [51,52]). Our approach to handling estimator bias is two-fold: First, we apply the bias-correction proposed in [53,54] and implemented in [55] to final AIS- and entropy-estimates. Second, we use non-parametric permutation testing [38,56] to test MI-estimates for statistical significance.

We here use permutation testing during the optimization as well as to test final AIS estimates. Permutation testing considers the MI-estimate as a test statistic in a test against the null-hypothesis of no relationship between the two variables, where the null-distribution is found through repeated estimation from permuted data [56]. Hierarchical permutation testing is applied during the optimization of the past state, $X_{t-1}^{-}$, by inclusion of past variables if they provide significant information about $X_{t}$. Note that, if no past variable provides information in a significant fashion, the inclusion terminates and no AIS is estimated. Hence, the proposed procedure tests for the presence of dependencies between past and current fixation and returns no estimate if no such dependency exists.

### 2.4. Experiment

As a proof of concept, we estimate AIS from eye tracking data recorded during a visual comparison task, where we varied observer states by adding a time constraint in one condition (see also [28] for details on the experimental setup).

#### 2.4.1. Participants

We recorded data from 13 participants (one female) with a mean age of 38, ranging from 21 to 53. Data of three participants had to be excluded from the analysis due to failures in the recording process, resulting in a sample of 10 all-male participants. All participants had normal or corrected to normal sight and gave their informed written consent before participating in the experiment.

#### 2.4.2. Task and Experimental Procedure

**Figure 2:**
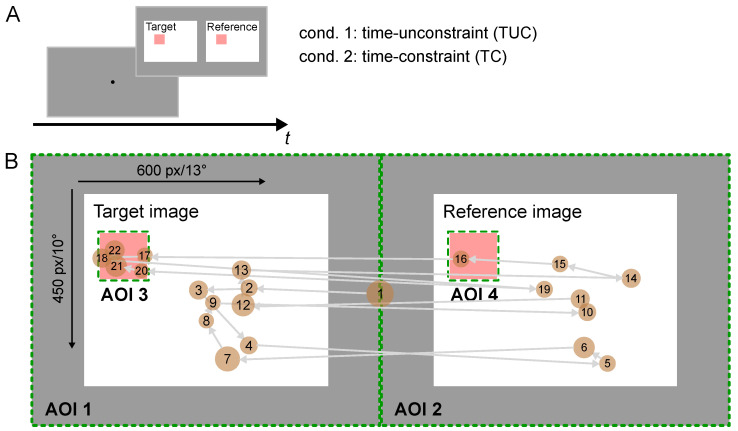
(**A**) Experimental setup for individual trial consisting of a screen showing the fixation dot and a screen displaying the image pair on a mean grey background. (**B**) Definition of areas of interest (AOI, green boundaries) on schematic images with target area (red box). The white line denotes an exemplary scanpath, where orange markers indicate ordered fixations and marker size corresponds to fixation time.

Participants were asked to identify as fast as possible the difference between a reference and a target image, where both images were identical except for one detail that was changed in the target image. Both images were presented next to each other on a mean gray background (Figure 2A). Participants were asked to indicate the location of the difference by clicking, using a regular computer mouse. The experiment took place in a quiet office environment under normal lighting conditions. Before the start of the experiment, participants were informed about the course of the experiment and received instructions.

Trials were recorded under two experimental conditions that were designed to induce two different user states, one relaxed state and one in which participants experienced stress through time pressure. Time pressure was achieved by varying the time available for the participants to complete the search task in each trial: In the first condition, participants had as much time as they needed (time unconstrained condition, TUC). In the second condition, time to finish the task was constraint to 9 s (time constrained condition, TC). The time limit was chosen such that it would lead to a significant performance drop and was determined in pre-tests. In addition, a sequence of nine accelerating tones, presented via headphones indicated the time running up in the TC condition. If participants did not find the difference between the images within the given time range, the next trial was initiated independently of the participant’s response. Performance dropped from 100% in the TUC condition to 65% correct trials on average in the TC condition with an average search time of m=4.84 s, sem=0.15 s (TUC: 100%, average search time: m=17.01 s, sem=1.5 s). This result indicates that the intended manipulation was indeed successful. All participants reported after the experiment that they felt under time pressure in the TC condition. For each condition, 22 trials were recorded. After half of the trials, participants were asked to take a break.

#### 2.4.3. Apparatus and Stimuli

Stimuli were presented on a Dell monitor. Participants saw 44 photographs of varying indoor and outdoor scenes. Images were taken from a publicly available database (Shuffle database, Large Change Images) [57], http://search.bwh.harvard.edu/new/Shuffle_Images.html. The experimental routine was programmed in Python using Psychopy [58,59]. The participants’ gaze behavior was recorded using a Pupil Labs eye tracker, using 120 Hz binocular gaze tracking and 60 fps world camera recordings [60].

The eye tracker was calibrated at the beginning of the experiment. All calibrations were done using the 9-point calibration routine implemented by Pupil Labs. Gaze points were mapped to the screen via the screen marker solution implemented by Pupil Labs. For that purpose, the monitor was defined as a surface based on 10 markers attached to the edge of the screen. To validate the calibration, participants were asked to fixate on a fixation dot presented at the center of the screen at the beginning of each trial (Figure 2A). The pupil labs eye tracker offers an accuracy of up to 0.6${}^{\circ }$ and a precision of 0.2${}^{\circ }$. If online-computed deviations between the recorded gaze position and the fixation dot exceeded 50 px (corresponding to a viewing angle of 1.15${}^{\circ }$), the eye-tracker was recalibrated.

Images presented during a trial categorized into easy, medium and difficult with respect to the search task prior to the experiment. Ratings were done by three experimenters independently resulting in 75% of all ratings to be 100% consistent, whereas for the remaining 25% (eleven images) of ratings deviated by one (e.g., easy, easy, and medium). To resolve these inconsistent cases, the median of the ratings was chosen as a label (e.g., easy). The image dataset was split in half, assuring an equal distribution of difficulty among the two. Half of the images were used for the TC condition (n=22) while the other half was used for the TUC condition (n=22). Within each condition, images were shown in a randomized order, with no image shown twice to the same observer.

#### 2.4.4. Preprocessing

Data analysis was done in Python and R [61]. Fixations for scanpath representations were computed using the basic Identification by Dispersion-Threshold (IDT) algorithm [62] using a maximum dispersion of 50 px and a minimum duration of 100 ms. Fixations above 1500 ms and data points with a confidence value below 0.9 were excluded from the data analysis. We analyzed data from all trials and did not differentiate between correct and incorrect trials.

Scanpaths were defined as sequential fixations of predefined areas of interest (AOI) and thus represent time-series data incorporating temporal as well as spatial information. We defined AOIs as four areas of interest (Figure 2B): (1) the left half of the monitor; (2) the right half of the monitor; (3) the target area in the left image; and (4) the respective target area in the right image. Target areas were defined based on the bounding boxes specifying the location of difference plus an additional frame of 50 px. Our approach aimed at extracting differences in the search process related to a presumably first “general search phase” and a “zooming in and validating phase” at the end of each trial.

## 3. Results

### 3.1. Optimization of Past States

**Figure 3:**
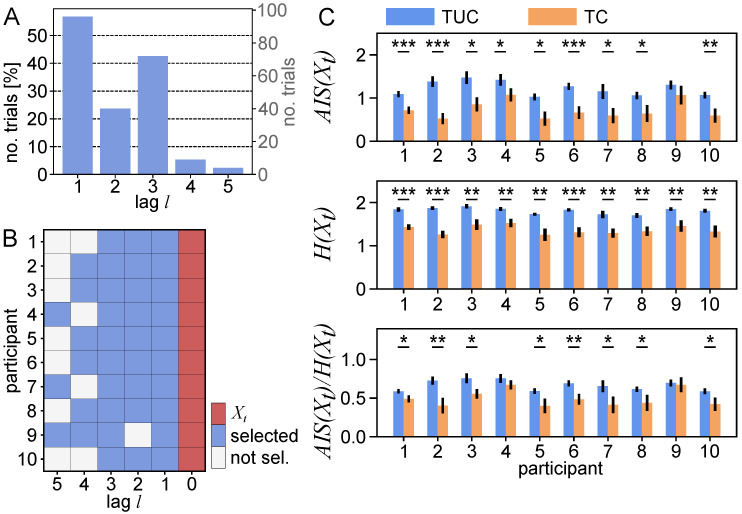
(**A**) Number of past variables with a given lag *l* selected through non-uniform embedding over participants and trials. (**B**) Union past state for each participant used for statistical testing. Selected past variables (blue) with lag *l* relative to the next fixation, $X_{t}$ (red). (**C**) Mean active information storage (AIS, top), entropy (middle), and normalized AIS (bottom), for conditions (*time constraint* (TC) and *time unconstrained* (TUC)) and individual participants (* p<0.05, ** p<0.01, *** p<0.001, error bars indicate the standard error of the mean).

We estimated AIS from scanpaths for each trial individually using the IDTxl Python toolbox [37]. We first optimized past states, $X_{t-1}^{-}$, while setting $k_{max}$ to 5 previous fixations. This resulted in a wide variety of selected past variables over trials and participants, where, in 74% of trials, variables with lags greater than one were selected (Figurer Figure 3A). Hence, in the majority of trials, fixations prior to the last fixation provided significant information about the next fixation and were relevant for quantifying the predictability of the scanpath. Furthermore, the variability in lags provides evidence for an intra- and inter-individual variance in viewing behavior that should be accounted for by estimation procedures.

### 3.2. Difference in Experimental Conditions

#### 3.2.1. Overall Effect of Condition on Predictability

To test for significant differences in predictability between the two experimental conditions, we fitted a linear mixed effects model with fixed effect *experimental condition* and random effect *participant*, allowing for a varying random slope for the effect of experimental condition on AIS values per participant [63]. For fitting the model, we used the lme4 package [64], written in R [61].

We found a main effect of experimental condition ($\chi^{2}\left(1\right)=30.054$, p<0.001), while we found no significant effect of the random slope. This indicates an overall effect of experimental condition on predictability when controlling for inter-subject variability. To assess how the experimental condition affected predictability for individual participants, we performed for each participant an independent samples permutation test between AIS in both conditions ($N_{perm}=5000$, Figure 3C). We found significantly decreased AIS in nine out of ten participants in the TC condition (p<0.05, $AIS_{TC}\left(X_{t}\right)<AIS_{TUC}\left(X_{t}\right)$).

#### 3.2.2. Relationship between AIS and Scanpath Entropy

In a second step, we investigated whether the decrease in AIS reflected a true decline in the predictability of the scanpath or whether it was rather due to a lower scanpath entropy in the TC condition. Since the absolute AIS value is bounded by the entropy of the two variables involved, a reduction in *absolute* AIS may not only be caused by a change in the predictability of a process, but also by a reduction in the processes’ entropy, i.e., a reduction in the information to be predicted.

We performed two-tailed, independent samples permutation tests for differences in $H(X_{t})$ between conditions for each participant ($N_{perm}=5000$, Figure 3), where we found a significant decline in $H(X_{t})$ for the TC condition for all participants (p<0.05, $H_{TC}\left(X_{t}\right)<H_{TUC}\left(X_{t}\right)$). To investigate if the decrease in $H(X_{t})$ may fully explain the decrease in $AIS(X_{t})$, we further tested for differences in AIS normalized by $H(X_{t})$, $AIS\left(X_{t}\right)/H\left(X_{t}\right)$. Here, we found a significant decline in eight out of ten participants (p<0.05, $AIS_{TC}\left(X_{t}\right)/H_{TC}\left(X_{t}\right)$ < $AIS_{TUC}\left(X_{t}\right)/H_{TUC}\left(X_{t}\right)$). This result indicates that the observed decrease in absolute AIS may be at least partially explained by a reduction in fixation entropy, but also by an actual reduction in the regularity or predictability of the scanpath.

Note that, for all statistical comparisons, to avoid spurious effects, we aimed at holding estimation bias constant between the groups to be compared. The estimation bias depends on the number of samples and the size of the variables used [51,54]. Hence, we fixed the number of samples by discarding samples at the beginning of a trial and created a uniform past state by taking the union of selected past states over all trials and conditions (Figure 3B). Taking the union ensures that the uniform past state contains all relevant variables at the expense of including potentially irrelevant variables in the estimation from some of the trials.

### 3.3. Decoding of Experimental Condition from Scanpath Predictability

It is commonly assumed that eye movements are partially modulated top-down as a function of task demand (e.g., [3,5,65]). Accordingly, in eye tracking analysis, a common objective is to determine if task or observer characteristics can be decoded from eye movements ([27,65,66], see also the work of Yarbus et al. for a classic example [67]). To evaluate whether AIS captures relevant information about the observer state induced by our experimental manipulation, we classified the experimental condition (TC versus TUC) from estimated AIS values per image, pooled over participants. Furthermore, we estimated GTE as the conditional entropy of the current fixation given the last, $H(X_{t}|X_{t-1})$, for each image and compared the classification performance using AIS as input feature to the classification performance using GTE as feature. We hypothesized that, if the long term information captured by AIS added significant information to the differentiation of the two experimentally induced observer states, then classification accuracy based on AIS should be higher compared to the classification accuracies reached by the GTE.

We followed the same classification approach as Borji and Itti [65], who successfully decoded different experimental tasks from fixation statistics recorded during viewing of images from two datasets (see also [66]). The authors used a random undersampling boosting algorithm (RUSBoost) [68] with decision trees as base models as well as a k-nearest neighbor classifier (KNN) with k=1. The RUSBoost classifier handles class-imbalances in the number of valid trials entering the analysis. The results are reported as the average classification accuracy over ten repetitions of a five-fold stratified cross validation. We found that the condition could be classified above chance level (0.6725) from AIS values using both the KNN (0.7369 ± 0.0535 SD) and RUSBoost classifier (0.6924 ± 0.0693 SD), where the highest accuracy was achieved using the KNN classifier. In comparison, classification from GTE resulted in a lower accuracy of 0.6870 (±0.0602 SD) for the KNN classifier and 0.6747 (±0.0647 SD) for the RUSBoost classifier. We performed a Wilcoxon signed-rank test on the difference in KNN classification performance and found that classification based on AIS as input feature was significantly higher than classification based on GTE (T=884.5, z=2.38, p<0.01). Our results demonstrate that AIS can be used successfully to decode our experimental condition from scanpath predictability, outperforming the classification based on the GTE, indicating that AIS captures more relevant aspects of the task-induced changes in observer state as encoded in eye movement behavior.

## 4. Discussion

We present AIS [35] as a novel approach to quantifying the predictability of scanpaths while accounting for long-range temporal dependencies between fixations. We demonstrated how to estimate AIS from scanpath data recorded during a visual comparison task and found that changes in observer states were reflected by changes in estimated AIS, indicating a lower predictability of gaze behavior in more demanding task conditions. Furthermore, we successfully decoded the experimental condition from AIS estimates of individual trials, achieving a higher accuracy compared to decoding the condition from the GTE, which was used in previous work to quantify scanpath predictability and identify differences in user states (e.g., [5]). We thus conclude that more relevant information about the observer state was encoded in the AIS estimate, which accounts for long-term dependencies in viewing behavior.

Current information-theoretic measures of predictability in scanpaths do not incorporate long-range temporal information, which may be important to accurately describe human viewing behavior [7,25,28,31,32]. Alternative measures, such as the SR model [25] or hidden Markov models [24,27], lack interpretability and their application is not always straightforward [5,25]. For example, the learning parameter representing the temporal horizon in the SR model has no clear interpretation, such that it is typically set through optimization of an additional criterion. Such an external criterion may not be readily available and may lead to circular analysis designs [69].

In contrast, AIS paired with novel estimation techniques, namely non-uniform embedding using a recently proposed estimation algorithm [36,37,38], allows optimizing the temporal horizon accounted for in a purely data-driven fashion. Furthermore, the optimized past state allows for a clear interpretation in units of past samples, i.e., fixations, offering additional explanatory value. Lastly, the past state is optimized individually per participant, accounting for inter-individual variation and including cases that are best modeled by a Markov chain of order one as a special case. In the latter case, i.e., if the optimized past state contains only the past fixation with lag one, AIS and GTE are complementary such that a change in GTE corresponds to an equivalent change in AIS and vice versa. When applying AIS estimation to scanpath data, we found significant temporal relationships in scanpaths beyond first-order transitions and high inter-individual variability. Both findings underline the importance of accounting for long-range temporal dependencies as well as inter-individual differences when modeling scanpath data, in particular when quantifying the regularity or predictability of gaze behavior.

As a proof of concept, we applied the AIS estimation to eye tracking data recorded from a visual comparison task, in which two different observer states were induced. In the TC condition, participants experienced a higher task demand compared to the TUC condition. Here, we found a significant decline in predictability measured by AIS for higher task demand. This result is in line with the majority of studies utilizing GTE, which find an increase in GTE and thus lower predictability under increased task difficulty (see [5] for a review). Furthermore, we were able to decode observer states from AIS and that decoding accuracy was higher for AIS than for GTE. We conclude that AIS is able to detect changes in predictability due to changes in task demand, while avoiding methodological ad hoc choices and being more versatile with respect to temporal correlations present in gaze behavior. Furthermore, we argue that AIS provides a more immediate measure of “predictability” as its calculation incorporates the *maximum* amount of information that can be gained from the past of a process about its next state [47,48]. Lastly, note that we here normalized our estimate of predictability by the entropy of the next fixation, also termed *stationary gaze entropy* [5]. We emphasize that such a normalization is necessary to exclude that changes in GTE or AIS are purely due to a change in fixation entropy between experimental conditions.

Being able to quantify changes in user states using approaches such as the one presented here, is central, for example, in many human machine cooperation scenarios [70]. Being able to detect user states and changes therein allows adapting machine behavior, e.g., to improve the interaction. Imagine for example a teaching assistance system, which, to provide optimal support for a student, must be able to assess whether a change in task demand, e.g., increasing the level of difficulty, is appropriate or overextending for the student. Only then the system can adjust to the right level of information supply or offer additional support for solving the task (see, e.g., [71]). For such an assessment of the human state, gaze behavior has been suggested as a rich data source, whose analysis can provide unobtrusive insights into a user’s cognitive or emotional state (e.g., [5,72,73]). For the analysis of gaze behavior, in particular information-theoretic measures have been suggested as promising markers of human states. We here extend existing work in this field by including previously neglected temporal correlations in the analysis of scanpath predictability, which may improve classification of user states, as demonstrated here by decoding the experimental condition from AIS. We therefore suggest AIS as an novel information-theoretic approach to the analysis of gaze behavior in user state modeling.

## 5. Conclusions

AIS is a promising measure for analyzing the predictability of scanpath data. Future work should extend its application to eye tracking data, for example, by exploiting the possibility to interpret AIS in a local (sample-wise) fashion [35]. Information-theoretic quantities such as entropy or mutual information allow for an interpretation for individual realizations of the random variables involved, which allows quantifying the local entropy or local predictability of a single fixation in time. Such a localized description of fixation sequences allows for a more fine-grained quantification of gaze behavior, up to the quantification of the predictability of a single fixation. Applying AIS in its localized version thus opens the possibility of using information-theoretic measures in real-time applications such as online monitoring or assistance (e.g., [11]). Furthermore, the application should be extended to other tasks, in particular free viewing, to gain further insights on how predictability changes as a function of the task at hand and given more natural viewing conditions. In addition, further comparison to existing scanpath analysis approaches in dedicated experiments are needed and are subject to future work. This will be a next important step to evaluate its potential for real-world gaze-based applications. In addition, note that our study is limited by its relatively small sample size and an all-male sample. Future research should therefore extend the application of AIS to larger and more diverse groups. Lastly, AIS estimation may be applied to non-discretized, raw (x,y)-fixation coordinates by foregoing the definition of AOIs and applying estimators for continuous data [74,75]. Further studies may explore these possibilities to further evaluate and extend the application of AIS to scanpath data. 

## Data Availability

The data presented in this study are available on request. The data are not publicly available because the original data contain sensitive information with the potential to identify study subjects and because authors lack participants’ consent for the publication of the data. Please direct any questions and data access requests to the Honda Research Institute Europe GmbH, Institutional Data Access/Ethics Committee contact: Julian Eggert (julian.eggert@honda-ri.de).

## Abbreviations

The following abbreviations are used in this manuscript:

AIS	Active Information Storage
GTE	Gaze Transition Entropy
IDTxl	Information Dynamics Toolkit xl
IDT	Identification by Dispersion-Threshold
MI	Mutual Information
SR Model	Successor Representation Model
